# Supplementary material for: Effects of particle size of ground alfalfa hay on caecal bacteria and archaea populations of rabbits
Source: PeerJ. 2019 Oct 18;7:e7910. doi: 10.7717/peerj.7910 (PMC6802586; doi:10.7717/peerj.7910)
Supplement: Table S1 [file peerj-07-7910-s001.docx]

**Table S1**

Diet composition and nutritional level

| Ingredients, % as fed | Particle size (µm) | | | |
| --- | --- | --- | --- | --- |
|  | 2500 | 1000 | 100 | 10 |
| Corn | 23.80 | 23.80 | 23.80 | 23.80 |
| Wheat bran | 29.10 | 29.10 | 29.10 | 29.10 |
| Soybean meal | 8.50 | 8.50 | 8.50 | 8.50 |
| Soybean oil | 1.00 | 1.00 | 1.00 | 1.00 |
| Alfalfa meal | 35.20 | 35.20 | 35.20 | 35.20 |
| Calcium carbonate | 0.05 | 0.05 | 0.05 | 0.05 |
| Calcium bicarbonate | 0.59 | 0.59 | 0.59 | 0.59 |
| L−Lysine | 0.10 | 0.10 | 0.10 | 0.10 |
| Choline chloride | 0.15 | 0.15 | 0.15 | 0.15 |
| DL-methionine | 0.13 | 0.13 | 0.13 | 0.13 |
| Sodium chloride | 0.40 | 0.40 | 0.40 | 0.40 |
| Mineral and vitamin premix^a)^ | 1.00 | 1.00 | 1.00 | 1.00 |
| Total | 100 |  |  |  |
| Chemical composition, g/kg DM |  |  |  |  |
| DM, % | 90.68 | 90.45 | 90.50 | 90.78 |
| Gross energy, MJ/kg of DM | 16.76 | 16.72 | 16.84 | 16.75 |
| Crude protein, % | 15.10 | 15.58 | 16.26 | 16.48 |
| Ether extract, % | 3.91 | 3.43 | 3.90 | 3.66 |
| Neutral detergent fiber, % | 28.98 | 29.03 | 29.25 | 28.26 |
| Acid detergent fiber, % | 15.85 | 17.78 | 14.68 | 15.73 |
| Ash, % | 6.76 | 6.91 | 6.71 | 6.83 |

^a^ The premix provided the following per kg of the diet: Vitamin A, 8000 IU; Vitamin D3, 1000 IU; Vitamin E, 2.5 mg; Vitamin K, 30.5 mg; Vitamin B6, 0.6 mg; Vitamin B12, 0.003 mg; Vitamin B1, 0.2mg; Vitamin B2, 1.6 mg; Folic acid, 0.05 mg; Nicotinic acid, 3.5 mg; Cu, 10mg; Zn, 50mg; Mn, 20mg; Fe, 50mg; Se, 0.1 mg; I, 0.5 mg; Robenidine, 100 mg.
