# Supplementary material for: Effects of particle size of ground alfalfa hay on caecal bacteria and archaea populations of rabbits
Source: PeerJ. 2019 Oct 18;7:e7910. doi: 10.7717/peerj.7910 (PMC6802586; doi:10.7717/peerj.7910)
Supplement: Table S2 [file peerj-07-7910-s002.docx]

**Table S2**

*P* value from PERMANOVA test of the differences in bacterial and archaeal community structures in rabbits cecum fed different particle size of alfalfa.

| Groups | | 10 µm | 100 µm | 1000 µm |
| --- | --- | --- | --- | --- |
| wei_unifrac |  |  |  |  |
| Bacteria | 2500 µm | 0.01 | 0.128 | 0.135 |
|  | 1000 µm | 0.001 | 0.989 |  |
|  | 100 µm | 0.012 |  |  |
|  | 10 µm |  |  |  |
| Archaea | 2500 µm | 0.0005 | 0.0005 | 0.025 |
|  | 1000 µm | 0.0008 | 0.0008 |  |
|  | 100 µm | 0.995 |  |  |
|  | 10 µm |  |  |  |
| unwei_unifrac |  |  |  |  |
| Bacteria | 2500 µm | 0.002 | 0.037 | 0.057 |
|  | 1000 µm | 0.009 | 0.868 |  |
|  | 100 µm | 0.009 |  |  |
|  | 10 µm |  |  |  |
| Archaea | 2500 µm | 0.003 | 0.003 | 0.042 |
|  | 1000 µm | 0.001 | 0.001 |  |
|  | 100 µm | 0.894 |  |  |
|  | 10 µm |  |  |  |

*p* < 0.05 is significant difference.
