# Supplementary figures and images for: Effects of particle size of ground alfalfa hay on caecal bacteria and archaea populations of rabbits
Source: PeerJ. 2019 Oct 18;7:e7910. doi: 10.7717/peerj.7910 (PMC6802586; doi:10.7717/peerj.7910)

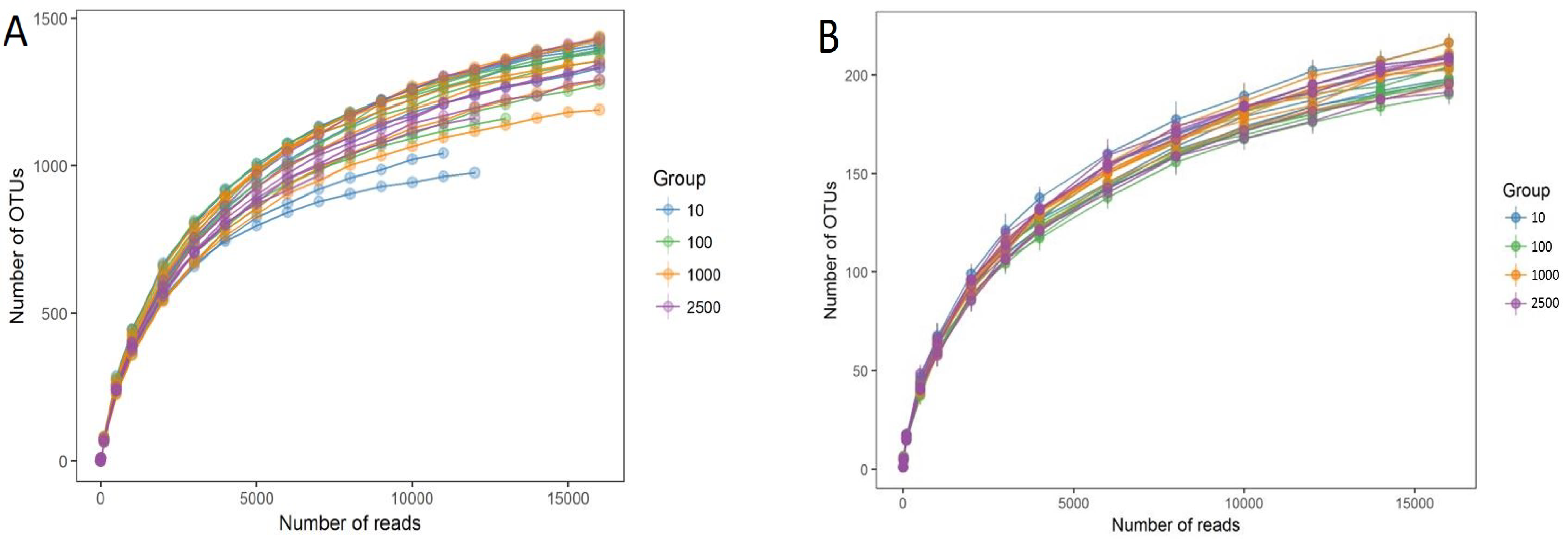

Supplement: Figure S1 [file peerj-07-7910-s003.png]

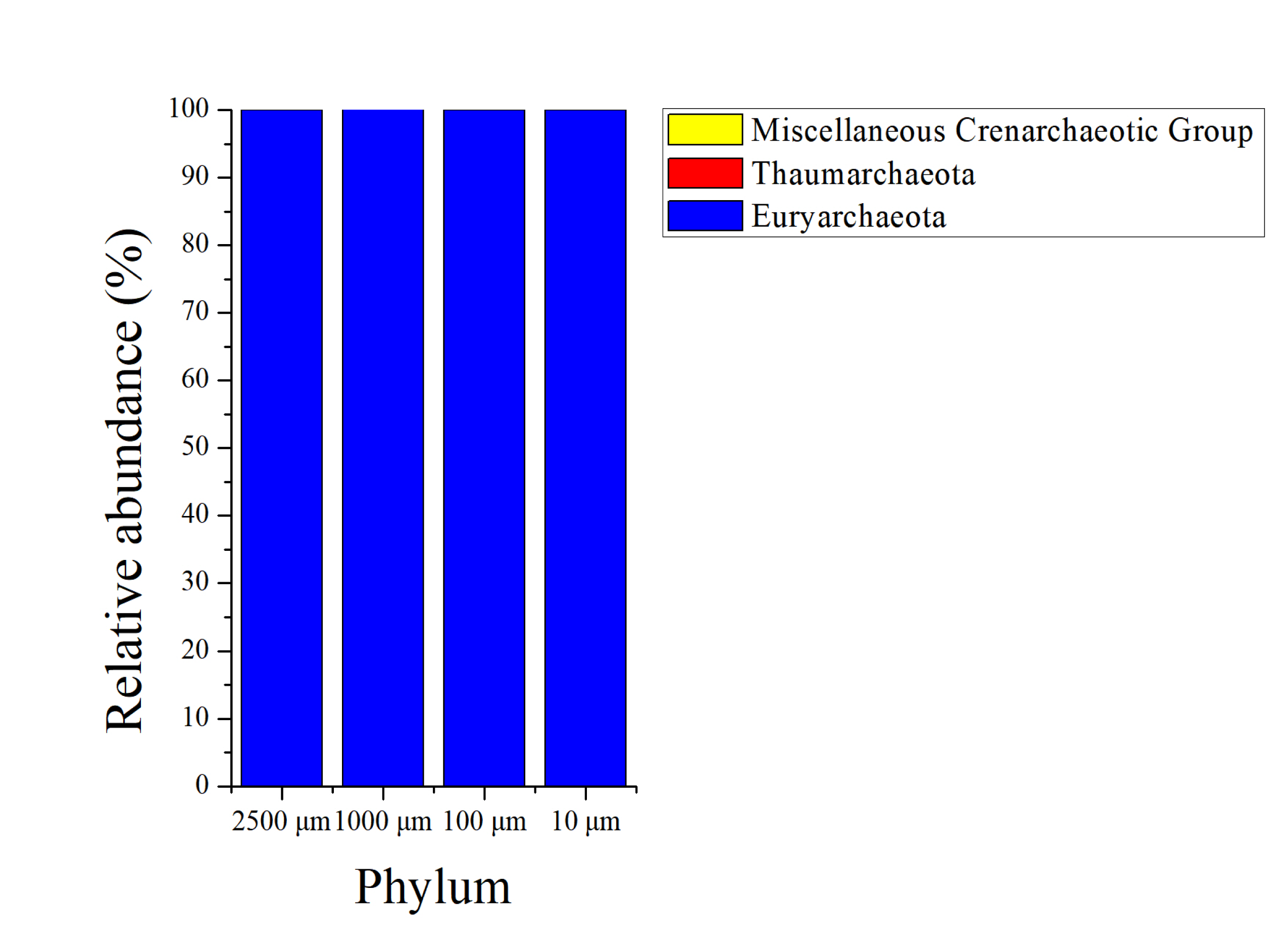

Supplement: Figure S2 [file peerj-07-7910-s004.png]

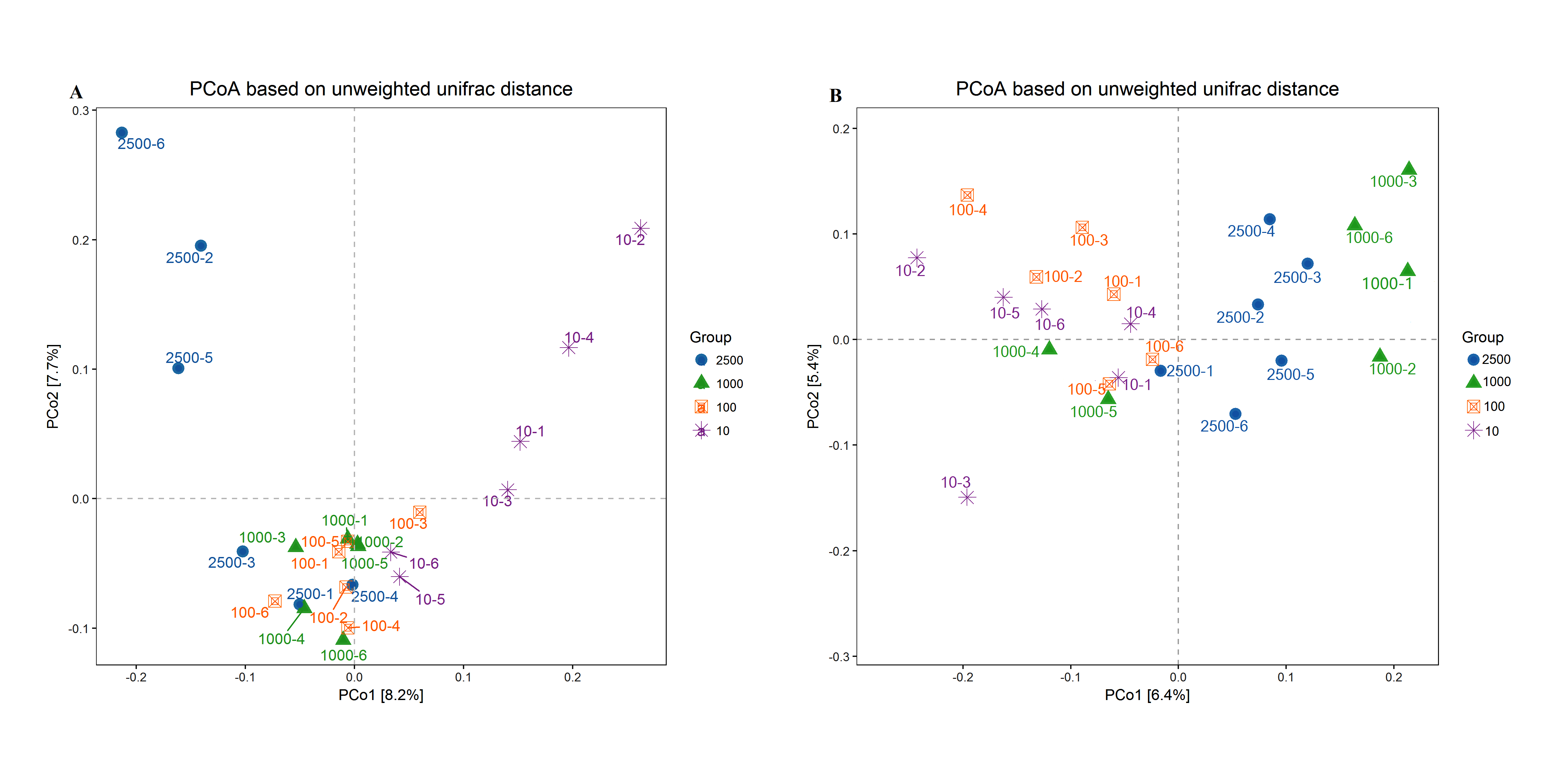

Supplement: Figure S3 — Principal co-ordinate analysis (PCoA) scores plot generated from rabbits caecum sample by a unweighted UniFrac analysis at the 97% similarity level. A and B represent bacteria and archaea, respectively. [file peerj-07-7910-s005.png]
